# Interactive Gene Expression Patterns of Susceptible and Resistant *Lens ervoides* Recombinant Inbred Lines and the Necrotroph *Ascochyta lentis*

**DOI:** 10.3389/fmicb.2020.01259

**Published:** 2020-06-24

**Authors:** Zhe Cao, Karan Kapoor, Li Li, Sabine Banniza

**Affiliations:** Crop Development Centre, Department of Plant Sciences, University of Saskatchewan, Saskatoon, SK, Canada

**Keywords:** RNA-seq, ascochyta blight, histopathology, weighed gene co-expression network analysis, differential gene expression

## Abstract

*Ascochyta lentis* is a foliar pathogen of *Lens* species and is of worldwide importance in cultivated lentil production. High levels of resistance were identified in the wild species *Lens ervoides*. This resistance was explored through histopathology, qPCR estimation of fungal biomass and transcriptome sequencing in a susceptible and a resistant recombinant inbred line (RIL) of *L. ervoides* infected with an aggressive isolate of *A. lentis*. Necrotrophic growth was delayed in the resistant RIL compared to accelerated necrotrophy of *A. lentis* in the susceptible RIL. Analysis of the fungal secretome indicated that the early activation of cell wall-degrading enzymes contributed to increased virulence of *A. lentis*. On the host side, gene co-expression analysis revealed that the invasion by *A. lentis* caused mRNA, DNA and protein decay in infected plants regardless of the level of resistance in the host. The resistant RIL exhibited a stronger gene co-expression in lipid localization and sulfur processes, and cellular responses to nutrients and stimuli than the susceptible RIL. In addition, differential gene analysis revealed that the repression of both, gibberellin signaling and cell death associated with the hypersensitive response (HR), were associated with enhanced *A. lentis* resistance.

## Introduction

Necrotrophic pathogens are the largest class of plant pathogens and responsible for major economic losses in a broad range of crops worldwide (Wang et al., 2014). The majority of necrotrophs exhibit facultative pathogenicity on their hosts by producing non-selective toxins that disrupt the normal function of affected cells (Mengiste, 2012). When necrotrophs infect plants in a conducive ambient environment, they actively secret a variety of pathogenicity agents such as cell wall degrading enzymes, phytotoxins, extracellular polysaccharides and other disruptive enzymes to break host plant cell walls, depolarize cell membranes, disrupt plant metabolisms, inhibit protein translation, or some or all of these combined to facilitate the expansion of necrosis (Laluk and Mengiste, 2010). To fend off such an attack, a plant must correctly recognize these pathogenic strategies and appropriately respond with its immune responses to attenuate disease symptoms.

The complexity of virulence mechanisms in necrotrophs is met with a sophisticated host immune systems, although our understanding of the molecular basis of resistance to necrotrophs is still limited. Briefly, plant immune systems consist of pathogen-associated molecular patterns (PAMPs) trigged immunity (PTI) and effector-triggered immunity (ETI) (Wang et al., 2014). PTI is the first phase of the innate immunity that is activated when PAMPs are perceived by plant receptors. The immune responses of PTI provide broad-spectrum and quantitative protection against a wide range of biotrophic, necrotrophic, and hemibiotrophic pathogens (Huang and Zimmerli, 2014). If pathogens overcome PTI, the second phase of defense, ETI, can be triggered through the recognition of pathogen effectors by effector-specific host disease-resistance (R) proteins, which results in a series of hypersensitive cell death responses (HR) at the colonization site (incompatible interaction) (Jones and Dangl, 2006). As biotrophic pathogens only thrive in living cells, it is believed that ETI is the widespread form of effective resistance against biotrophs. In contrast, several studies have indicated that ETI can facilitate the invasion of host plants by necrotrophic pathogens and increase disease severity due to the proliferation of necrotrophs in dead host cells (Coll et al., 2011). Indeed, some necrotrophs such as *Cochliobolus victoriae* (causal agent of victoria blight) in *Arabidopsis* (Lorang et al., 2007) and *Botrytis cinerea* (causal agent of gray mold) in tomato (El Oirdi and Bouarab, 2007) were shown to hijack the plant immune systems and use the host HR machinery to boost their virulence. Hammond-Kosack and Rudd (2008) introduced a “susceptibility signaling” model to explain how the necrotrophic pathogen *Mycosphaerella graminicola* successfully induces HR-related susceptibility in wheat by secreting effectors specific to host receptors. A response strategy of plants is to alleviate or prevent such HR-triggered programmed cell death (PCD). In tobacco, Rossi et al. (2017) found that the expression of anti-PCD compounds was effective against the necrotrophic pathogen *B. cinerea*. Similarly, Chowdhury et al. (2017) observed that reactive oxygen species (ROS) signaling which promotes PCD was downregulated to confer enhanced resistance to the necrotrophic phase of the hemibiotrophic pathogen *Macrophomina phaseolina*. However, it is still not clear if these strategies can be extended to all necrotrophs, as some studies reported that the increased resistances toward necrotrophs could be achieved by upregulation of R-genes and elevated salicylic acid (SA) levels which are two major components accounting for HR aggregation (Zhu et al., 2017; Kouzai et al., 2018).

With the recent advances in new generation sequencing, high throughput transcriptome sequencing (RNA-seq) has been employed to understand disease responses in a broad range of plant species (Becker et al., 2017; Cao and Deng, 2017; Cui et al., 2017; Khorramdelazad et al., 2019). Weighed gene co-expression network analysis (WGCNA) is one powerful method to analyze high dimensional sequencing data across treatments, time points and genotypes (Oldham et al., 2006; Greenham et al., 2017). WGCNA includes estimation of gene connectedness and the construction of scale-free networks, all of which can facilitate the identification of important regulatory networks and genes (hub genes) (Langfelder et al., 2011). Another useful method for transcriptome investigation is the differential gene expression (DGE) analysis that relies on the statistics of pair-wise comparisons of gene expression to identify the differences in the transcription between different treatments. Both approaches have been frequently adopted in analyzing transcriptomes (Ficklin and Feltus, 2011; Weston et al., 2011; Pérez-Delgado et al., 2016; Dinkins et al., 2017).

The success of a transcriptome experiment investigating the interaction of a pathogen and its host plant highly depends on the appropriate sampling time. Microscopic histopathology is the most commonly adopted method that reveals the dynamic growth of pathogens in their hosts. This method provides opportunity for quantitative assessment of germination, penetration and proliferation of fungal structures in the host cells. However, determination of fungal growth through microscopy is laborious, and the results of visual quantification can be subjective (Tellenbach et al., 2010). An alternative approach to assess fungal growth is based on quantifying the ratio of fungal gDNA to that of its host by quantitative PCR (qPCR). This method is high-throughput and produces objective assessments of fungal biomass, but does not provide information on specific fungal developmental stages. Several studies on a range of host-pathogen systems showed that these two methods complement each other well in assessments of fungal growth (Tellenbach et al., 2010; Horevaj et al., 2011; Weβling and Panstruga, 2012).

Here, we recruited these tools to study transcriptomes of *Lens ervoides* in response to attack by the necrotrophic pathogen *Ascochyta lentis* that causes the economically important foliar disease ascochyta blight on cultivated lentil (*Lens culinaris* spp. *culinaris*) (Rubiales and Fondevilla, 2012). Lentil accounted for an estimated global production of 7.6 million tons in 2017 (FAOSTAT, 2017) and represents an important source of protein in many parts of the world. Due to limited genetic variability in the gene pool of cultivated lentil, the search for enhanced resistance to *A. lentis* (and other pathogens) has been focused on wild relatives including *L. ervoides*, and germplasm highly resistant to *A. lentis* was identified (Bhadauria et al., 2017; Sari et al., 2017). Studying an intraspecific F_9_ recombinant inbred line (RIL) population of *L. ervoides*, transgressive segregants that displayed contrasting ascochyta blight resistance were identified, indicating the potential for genetic variability in resistance that could be further explored (Bhadauria et al., 2017).

Our objective was to elucidate the genetic mechanism of resistance to *A. lentis* by contrasting the interactions of this pathogen with a resistant and a moderately susceptible *L. ervoides* RIL. Relevant time points for transcriptome sequencing were determined by performing histopathological and qPCR studies to quantify the fungal growth during the first 14 days after inoculation. For transcriptomic data analysis, we separated transcriptomes between plant and pathogen as a first step, and then used WGCNA or DGE analysis to identify important regulatory networks, pathways or genes from these two sets of transcriptomes. The identification of significant and variable gene reprogramming between the resistant and susceptible *L. ervoides* RILs, and between *A. lentis* invading these two RILs, could extend our knowledge of how plants effectively use their immune systems to defend themselves from necrotrophic pathogens.

## Results

### Histopathological and qPCR Quantification of *A. lentis* Growth Between RILs

**FIGURE 1:**
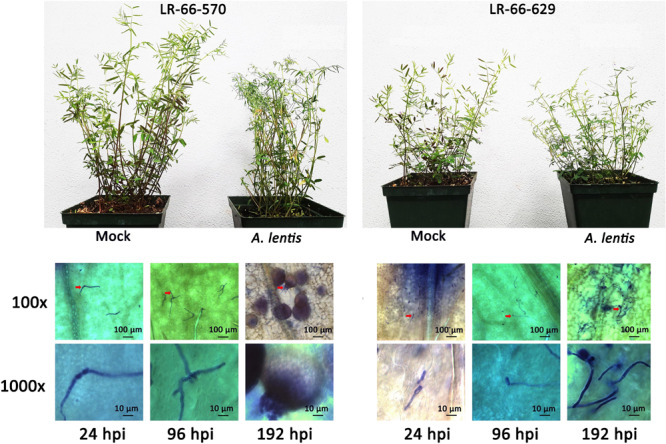
Levels of *Ascochyta lentis* resistance in the susceptible *Lens ervoides* RIL LR-66-570 **(top left)** and resistant RIL LR-66-629 **(top right)** at 192 h after *A. lentis* inoculation compared to mock-inoculated plants. Progression of *A. lentis* at the cellular level (100x and 1,000x magnification) in RIL LR-66-570 **(bottom left)** and RIL LR-66-629 **(bottom right)** at 24, 96, and 192 h post inoculation.

**FIGURE 2:**
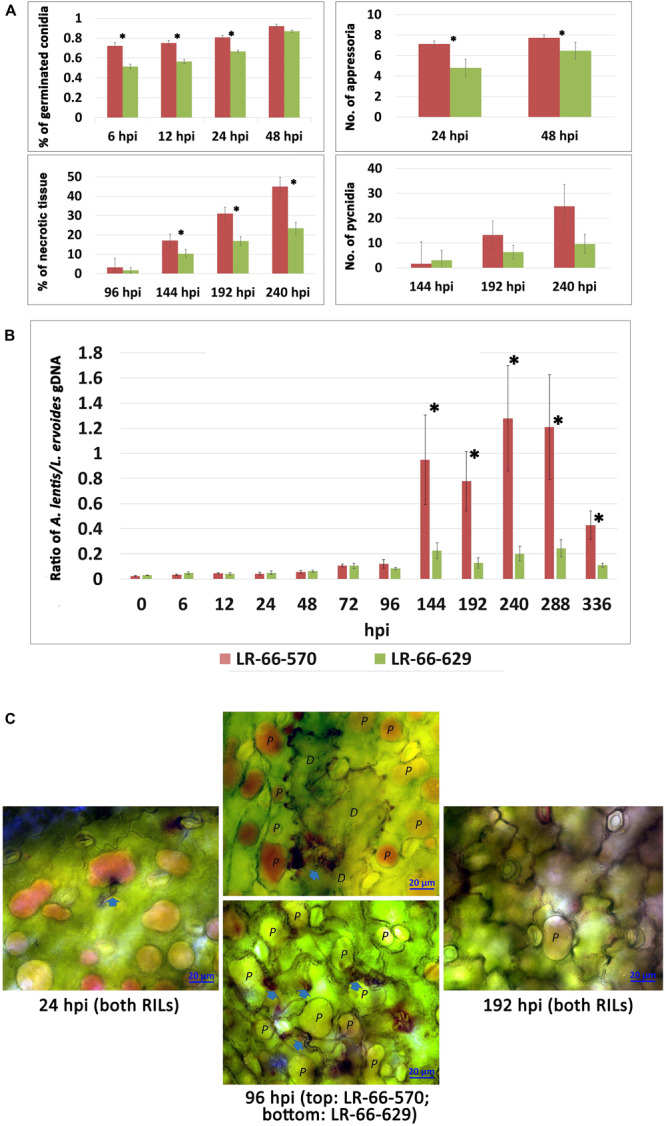
Quantitative assessment of *Ascochyta lentis* progression in susceptible *Lens ervoides* RILs LR-66-570 and resistant LR-66-629. **(A)** Histopathological assessment of four *A. lentis* developmental parameters in two RILs from 6 to 240 h post inoculation (hpi). **(B)** qPCR quantification of relative *A. lentis* biomass in the two RILs from 0 to 336 hpi. The *A. lentis* biomass was determined as the gDNA ratio between *A. lentis* (estimated based on β-tubulin) and *L. ervoides* (estimated based on elongation factor 1α). Asterisks indicate significant differences between means at *P* < 0.05. Error bars represent standard errors of the means. **(C)** Changes in viability of epidermal cells at 24, 96 and 192 hpi with *A. lentis*. Photos reflected the most prevalent events in RILs. Viable cells (labelled as P) show accumulation of the dye in the vacuole only whereas dead cells (labelled as D) are stained throughout.

The inoculation experiment revealed distinct differences in the level of resistance to *A. lentis* between *L. ervoides* RILs LR-66-570 (susceptible) and LR-66-629 (resistant) (Figure 1). Conidial germination of *A. lentis* was recorded at 6, 12, 24, and 48 hpi and was statistically higher on the susceptible RIL LR-66-570 than on the resistant RIL LR-66-629 during the first 24 h, whereas it was similar on both RILs at 48 hpi (Figure 2A). Likewise, the number of appressoria differentiating from germ tubes of germinated conidia on LR-66-570 was significantly higher than that on LR-66-629 during the period from 24 to 48 hpi. As leaf lesions could be first discerned at 96 hpi, we decided to estimate the area (%) of necrotic leaf tissue from 96 to 240 hpi in 48-hour-intervals. The area of necrotic tissue increased as incubation time increased, and starting at 144 hpi LR-66-570 displayed significantly more leaf injuries than LR-66-629. As a polycyclic disease with repeated cycles of conidia production, the number of pycnidia produced on the plant will determine the rate of disease increase. Although not statistically significant, estimates of the number of pycnidia on both RILs indicated a trend for on average two- to three-times more pycnidia on LR-66-570 than on LR-66-629 at 192 and 240 hpi. Subsequently, we used qPCR with fungal and plant specific primers to estimate the relative fungal gDNA in infected leaves (Figure 2B). Results showed that in both RILs, fungal growth was very limited during the first 96 hpi, but then increased dramatically in LR-66-570 and was significantly higher than in LR-66-629. Taken together, these observations suggested that (1) the growth of *A. lentis* in LR-66-570 was more aggressive than in LR-66-629, and (2) the growth of *A. lentis* could be divided into three different stages: the germination and penetration stage (0–48 hpi), early colonization (48 to 96 hpi), and necrotrophic stage (appearance of necrotic tissues) (after 96 hpi). To gain insight into responses of plant cells to infection by *A. lentis*, we then performed viability test for both RILs at 24, 96, and 192 hpi that represented these three *A. lentis* growth stages (Figure 2C). Results showed that cell death rarely occurred in either RIL during the penetration stage at 24 hpi. When infection had progressed for 96 hpi, we frequently observed cell death around infection sites in LR-66-570, whereas this was not commonly seen in LR-66-629 whose cells were largely viable in colonized tissues. These results indicate that the switch between intercellular colonization and necrotrophy probably occurred around 96 hpi in the resistant RIL, but started earlier in the susceptible RIL. At 192 hpi, cell death was common around the infection sites in both RILs, indicating that *A. lentis* infection had proceeded to the necrotrophic phase in both RILs. Based on these results, sampling time points of 24, 96, and 192 hpi were selected for transcriptome sequencing.

### Simultaneous Assembly of Plant- and Pathogen-Specific Transcriptomes

**FIGURE 3:**
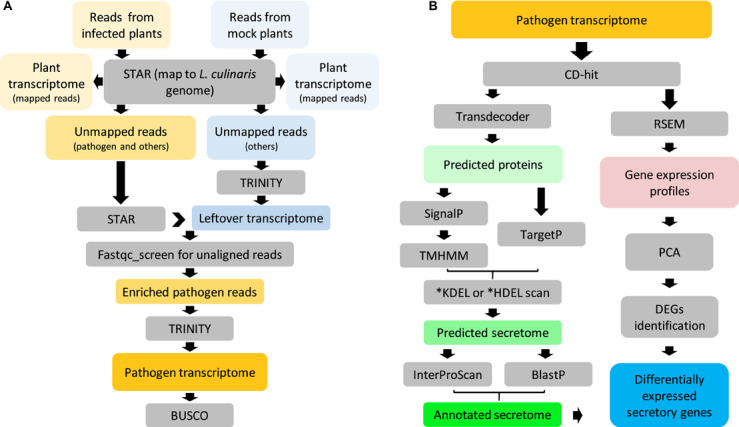
Overview of *Ascochyta lentis* sequencing data analysis. **(A)** Enrichment of *A. lentis* reads from mixed reads for *de novo* transcriptome assembly. **(B)** Prediction, annotation and expression profile of *A. lentis* secretome.

Transcriptome sequencing was performed on both, uninfected and infected samples. With the aid of the *L. culinaris* reference genome V1.2 (Bett et al., 2016), it was then possible to assemble plant- and pathogen-specific transcriptomes simultaneously. Plant transcriptomes consisted of reads (76–93%) that were uniquely mapped to the reference genome (Supplementary Figure S1). A small proportion of unmapped reads (2–6%) containing pathogen reads were processed to enrich pathogen reads only, which resulted in 3.6 M pair-ended reads (≈494 MB) that likely belonged to *A. lentis* (Figure 3A). *De novo* assembly using those reads yielded a transcriptome that comprised 34,565 “Trinity genes” corresponding to 35,878 transcripts (Table 1). We then assessed the transcriptome completeness of *A. lentis* using Benchmarking Universal Single-Copy Orthologs (BUSCO) by comparing all *A. lentis* transcripts with the BUSCO content of the Ascomycota division to which *A. lentis* belongs. Among 1,315 BUSCO genes, 1,228 BUSCOs, including 985 complete and 243 partial BUSCOs, were successfully retrieved, which indicated that only 87 BUSCOs were missed and that the *de novo* assembled *A. lentis* transcriptome possessed a completeness of 93.5%, thus was of sufficiently high integrity for further studies.

**TABLE 1:** Descriptive statistics of *de novo* assembly of the *Ascochyta lentis* transcriptome.

Metric	Value
**Overall assembly**	
Total trinity “genes”	34,565
Total trinity transcripts	35,878
Percent GC	53.47
**Contigs**	
Contig N10	2,797
Contig N20	2,129
Contig N30	1,751
Contig N40	1,414
Contig N50	1,107
Median contig length	398
Average contig	688
Total assembled bases	24,710,864
**Longest isoform per gene**	
Contig N10	2,760
Contig N20	2,091
Contig N30	1,722
Contig N40	1,379
Contig N50	1,076
Median contig length	397
Average contig	674
Total assembled bases	23,323,748
**Ascomycota BUSCO genes**	
Complete and single-copy BUSCOs (S)	933
Complete and duplicated BUSCOs (D)	52
Fragmented BUSCOs (F)	243
Missing BUSCOs (M)	87
Total BUSCO groups searched	1,315
Total BUSCO groups retrieved	93.5%

### Annotation and Expression Profile of the *A. lentis* Transcriptome

The pipeline for the analysis of the *A. lentis* transcriptome is shown in Figure 3B. After removal of redundancy, the *A. lentis* transcriptome was submitted to TransDecoder-based prediction of open reading frames, which resulted in the successful prediction of 19,834 putative coding transcripts (Supplementary Table S2). Among these coding transcripts, 1,139 ORFs were predicted to possess signal motifs. After excluding those sequences with transmembrane domain(s) or C-terminal motifs of KDEL or HDEL, 904 remaining ORFs were recognized as putative secretory proteins.

### Gene Expression in RILs and the Pathogen Followed Different Temporal Dynamics During Infection

**FIGURE 4:**
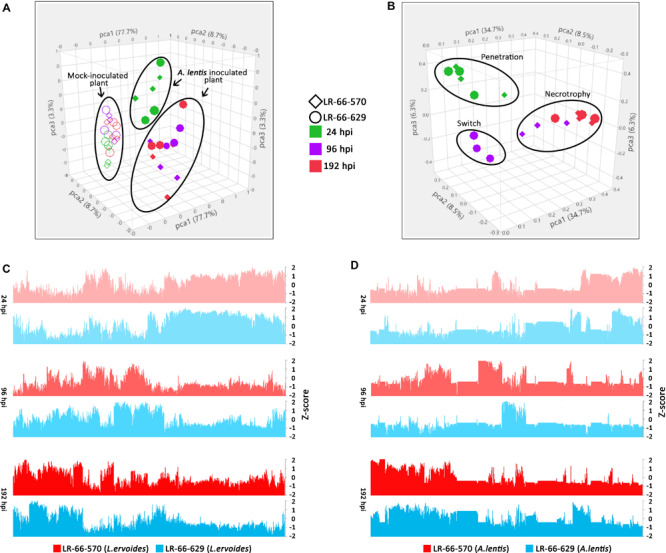
Overview of transcriptome variability of *Lens ervoides* RILs LR-66-629 (resistant) and LR-66-570 (susceptible), and *Ascochyta lentis* samples at 24, 96 and 192 h post inoculation (hpi). Each point in the PCA plot represents an individual sample. **(A)** PCA clustering of host samples. Mock-inoculated samples are indicated by open symbols and *A. lentis*-inoculated samples are depicted by solid symbols. **(B)** PCA clustering of pathogen samples. Samples collected at 24 (green), 96 (purple) and 192 (red) hpi from LR-66-570 (circle) and LR-66-629 (diamond) were used for analysis. **(C)** Overview of gene expression for *L. ervoides* samples. **(D)** Overview of gene expression for *A. lentis* on two *L. ervoides* hosts. The normalized value (*Z*-score) was used to describe the gene expression for each sample at 24, 96, and 192 hpi.

To evaluate the variability among transcriptomes of *L. ervoides* RILs and *A. lentis* samples, we conducted PCA to reduce the data dimensionality for ease of assessment (Figures 4A,B). Within the *A. lentis*-inoculated group, samples were mainly separated based on whether they were inoculated or not, and partly based on incubation time, as mock-inoculated samples segregated from *A. lentis*-inoculated samples (Figure 4A). Inoculated samples collected at 24 hpi were separated from samples analyzed at 96 and 192 hpi, whereas differences between the RILs were less obvious. These results indicated that the infection of *A. lentis* induced striking plant transcriptome responses and such responses largely varied between early and late incubation time. In contrast to RILs, the PCA plot showed that the transcriptome of *A. lentis* responded quite distinctly to the two RILs (Figure 4B). We found that the *A. lentis* transcriptomes in the susceptible LR-66-570 were separated into two groups, with one at 24 hpi, and the other at 96 and 192 hpi, which was different from the resistant LR-66-629 where three groups corresponding to 24, 96, and 192 hpi could be distinguished.

We also produced bar plots to describe the gene expression for infected plants (Figure 4C) and pathogen samples (Figure 4D). The transcriptional behavior of host genes largely followed a temporal trend, which was consistent with what was observed in PCA. For the pathogen, we found that the gene expression of *A. lentis* was similar in both hosts at 24 and 196 hpi, but was distinctly different in the resistant LR-66-629 from that in the susceptible LR-66-570 at 96 hpi (Figure 4D). This corresponded to histopathological findings indicating that *A. lentis* underwent penetration into the host tissue at 24 hpi and was in the necrotrophic phase at 192 hpi in both RILs, but appeared to progress to the necrotrophic phase early in the susceptible LR-66-570 as evident by extensive cell death at 96 hpi.

### *A. lentis* Accelerated Host Cell Wall Degradation, Host Cell Adhesion and Induction of PCD in the Susceptible *L. ervoides* RIL

**FIGURE 5:**
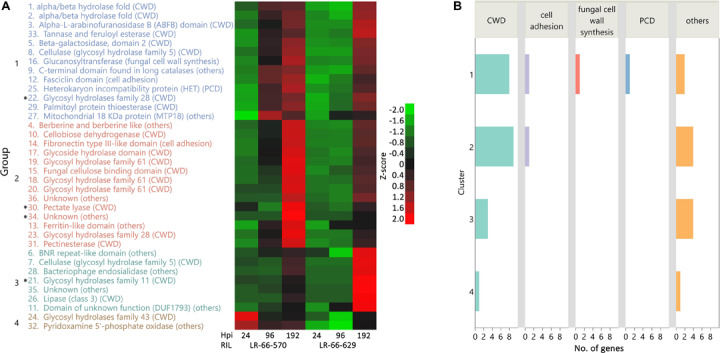
Time-course dynamics of differentially expressed *Ascochyta lentis* secretory genes in the two *Lens ervoides* RILs LR-66-629 (resistant) and LR-66-570 (susceptible). **(A)** Hierarchical clustering of 36 secretory genes (Ward’s method) for two RILs at 24, 96 and 192 h post inoculation (hpi). Genes with the same color are from the same group. Genes with asterisks are predicted effectors. **(B)** Functional classification of secreted genes among clusters.

Pathogen-secreted proteins play crucial roles in host colonization and expansion during the necrotrophic phase of infection (Laluk and Mengiste, 2010). To understand how *A. lentis* employed these proteins in the resistant and the susceptible RILs over time, we first compared the gene expression of *A. lentis* in the two RILs, which resulted in the identification of 944 fungal DEGs. Among these 944 fungal DEGs, 36 *A. lentis* secretory genes were identified (Supplementary Table S3). According to their expression patterns, these 36 genes were hierarchically clustered into four different groups (Figure 5A). Among them, groups 1 and 2 contained 27 genes that were expressed at a higher level in the susceptible LR-66-570 than in the resistant LR-66-629 at either 96 or 192 hpi. Group 3 comprised seven genes that had higher expression in LR-66-629 than on LR-66-570 at 192 hpi. Two genes in group 4 were highly expressed in LR-66-570 at 24 and 96 hpi when compared with their expression in LR-66-629.

Based on current knowledge, these secretory proteins were grouped into the biological functions of cell wall degradation (21 genes), fungal cell wall synthesis (1 gene), induction of PCD (1 gene), cell adhesion (2 genes), and others (12 genes) (Figure 5B). It was evident that a larger proportion or all *A. lentis* genes that regulated cell wall degradation (18/21 genes), cell adhesion (2/2 genes), fungal cell wall synthesis (1/1), and induction of PCD (1/1) were expressed at higher levels in the susceptible LR-66-570 than in LR-66-629 at 96 (group 1), 192 hpi (group 2) or at all time points (group 4). Among these secretory genes, four genes were predicted to be effectors, including two genes coding for glycosyl hydrolases (group 1 and 3), one gene coding for a pectate lyase (group 2) and another one gene coding for a protein without a clear function (group 2).

### Consensus Gene Co-expression Network Analysis Revealed Common Gene Co-regulation Relationship Between *L. ervoides* RILs

**FIGURE 6:**
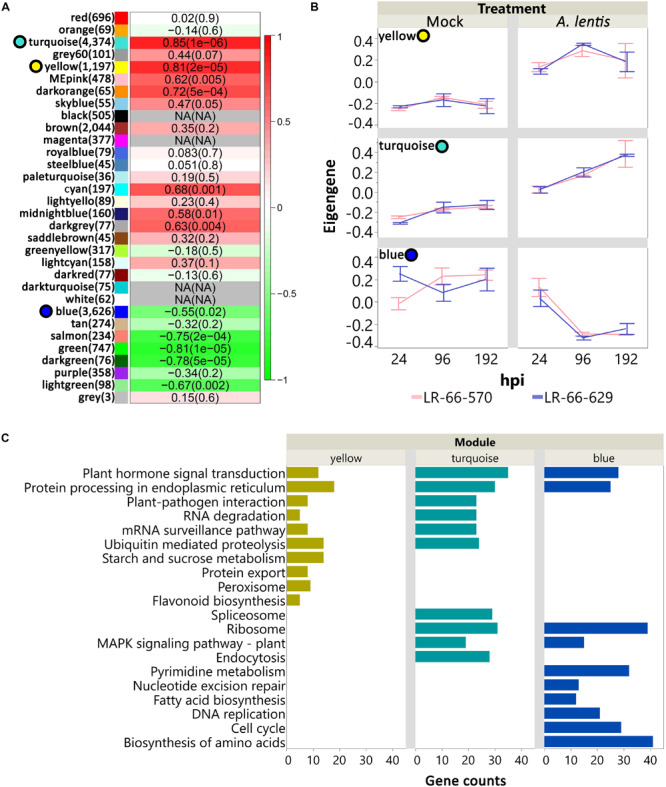
Analysis of consensus networks of gene expressions in mock or *Ascochyta lentis*- infected tissues of *Lens ervoides* RIL LR-66-570 (susceptible) and LR-66-629 (resistant). **(A)** Correlation between module eigengenes and treatment (mock or *A. lentis*-inoculated). Modules are arbitrarily identified by color names. Number of genes for each module is shown in parentheses to the right of the module name. Correlation coefficient and *P* value for each module is shown inside of heatmap. A *P* value cutoff of 0.01 was set to declare significant modules. Top three significant, major modules (yellow, turquoise, blue) are highlighted with colored circles to the left of module name. **(B)** Time-series gene expression of yellow, turquoise, and blue for mock and *A. lentis*-inoculated RILs. Value of eigengene represents the overall gene expression of that module. Error bars represent standard errors of the means. **(C)** Top significant KEGG terms enriched in yellow, turquoise, and blue modules. The vertical axis indicates the significant KEGG terms and horizontal axis represents gene counts for each KEGG term. An adjusted *P* value of less than 0.05 was set to declaring significant KEGG terms.

Principal component analysis showed that a large proportion of genes in LR-66-629 and LR-66-570 appeared to have similar transcription dynamics in response to the challenge with *A. lentis*. To further discern these dynamic responses, we first employed a weighed gene co-expression method. Results revealed a consensus gene co-expression network consisting of 32 modules based on a total of 16,786 genes (TMM > 25) and 36 libraries. The density (D) value of 0.85 indicated that the gene co-expression relationships of these modules (identified by randomly assigned colors) were highly conserved between RILs (Supplementary Figure S2). To identify which modules significantly responded to *A. lentis* infection, we correlated the module eigengenes with the binary data of “0” for mock and “1” for *A. lentis* inoculation. Of these modules, 12 modules (11,589 genes) displayed significant correlations (*P* < 0.01) with binary data (Figure 6A). Three of the 12 were major modules (78%, 9,197 out of 11,589 genes), including yellow (1,197 genes), turquoise (4,374 genes), and blue (3,626 genes). These major modules appeared to play more important roles in global transcriptome regulations than other minor modules. To summarize gene expression dynamics of these modules, we plotted their eigengenes over time for the two RILs for mock and *A. lentis-*inoculated samples (Figure 6B and Supplementary Table S4). Both RILs displayed similar expression patterns among all modules, with the exception of mock-inoculated samples in blue. Inoculation with *A. lentis* resulted in upregulation of the gene expression in yellow and turquoise, whereas gene expression was downregulated in blue. To further understand which biological pathways were involved in those modules, the genes with known *Medicago truncatula* orthologs were mapped to the KEGG database (Figure 6C). The results showed that several pathways were commonly enriched in all three modules, such as “Plant hormone signal transduction” and “Protein processing in endoplasmic reticulum,” whereas others were up- or downregulated during the *A. lentis* infection, depending on the module. In yellow and turquoise, the presence of “Plant-pathogen interaction,” “RNA degradation,” “Ubiquitin mediated proteolysis” pathways and “mRNA surveillance pathway” suggested that genes within those pathways were upregulated after the challenge with *A. lentis*. Six pathways, “Pyrimidine metabolism,” “Nucleotide excision repair,” “Fatty acid biosynthesis,” “Mismatch repair,” “DNA replication,” “Cell cycle,” and “Biosynthesis of amino acid,” were specifically enriched in blue, indicating that genes within these pathways were downregulated after *A. lentis* infection.

### Differential Gene Co-expression Analysis Between RILs

**FIGURE 7:**
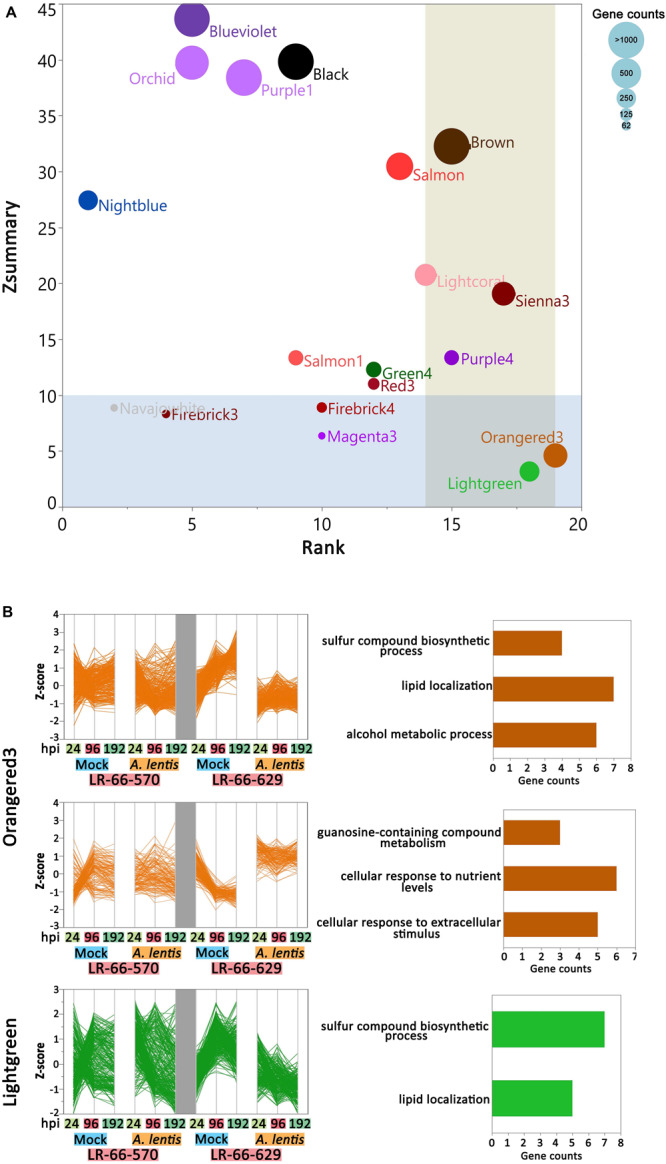
Differential co-expression in the resistant *Lens ervoides* RIL LR-66-629 with respect to the susceptible RIL LR-66-570 after inoculation with *Ascochyta lentis*. **(A)** Module preservation statistics of Zsummary (vertical axis), Median Rank (horizontal axis) and module sizes (proportional size of bubbles) among 19 constructed modules. Blue shaded plot area represents Zsummary value < 10, beige shaded plot area represents Medium Rank > 14. Modules falling into both shades were declared modules with low preservation between RILs. **(B)** Expression patterns and top enriched Gene ontology (GO) terms for networks with low preservation between RILs. Hierarchical clustering (Ward’s method) analysis was performed to visualize the gene expression patterns in Orangered3 and Lightgreen modules. GO terms are displayed to the right of modules.

To assess if the within-module topology was consistent between the susceptible RIL LR-66-570 and the resistant RIL LR-66-629, modules identified in the LR-66-629 network were assessed for preservation level in the LR-66-570 network (Supplementary Table S5). According to the criteria proposed by Langfelder et al. (2011), Lightgreen and Orangered3 (Figure 7A) that possessed low Zsummary scores and medium Rank values were declared as modules with low preservation between RILs. As this test was centered on LR-66-629, i.e., module assignments in LR-66-570 were derived from the structure of the LR-66-629 gene co-expression network, gene co-expression trends of Orangered3 and Lightgreen were evident in LR-66-629, but ambiguous in LR-66-570 (Figure 7B). To identify the biological relevance of the two modules, genes with *Medicago truncatula* orthologs were mapped to the Gene Ontology (GO) database (Figure 7B). Results revealed that those genes downregulated after *A. lentis* infection in Lightgreen and Orangered3 were commonly enriched in “sulfur compound biosynthesis process” and “lipid localization” pathways. Other genes that were upregulated upon *A. lentis* invasion in Orangered3 were significantly enriched in “guanosine-containing compound metabolic process,” “cellular response to nutrient levels,” and “cellular response to extracellular stimulus.” By analyzing a gene network table we then identified ten highly connected genes (weight cutoff > 0.20) as the hub genes in each module, among which four were involved in PCD, four in cell wall modulation, two in SA signaling, one in ROS, and eight genes with unknown roles in plant defense (Supplementary Figure S3).

### DGE Analysis Indicated HR and PCD Were Significantly Promoted in LR-66-570

As the susceptible RIL LR-66-570 and the resistant RIL LR-66-629 are genetically distinct, they may have differentially expressed genes (DEGs) that correlate with plant characteristics other than disease defense responses. To eliminate this background noise, it was necessary to limit the DGE analysis to those genes that are involved in disease defense responses only. Therefore, we performed pair-wise comparisons between pathogen inoculated plants and mock-inoculated plants for each genotype at 24, 96, and 192 hpi, which resulted in the observation of 11,653 disease-responsive genes whose expression levels were significantly influenced by pathogen inoculation. Of those genes, 1,307 genes displayed significant difference in their expression levels between RILs and were regarded as DEGs (Supplementary Table S6). Among them, 10 genes upregulated in LR-66-629 and another 10 genes upregulated in LR-66-570 were validated by qPCR quantification in an independent inoculation experiment (Supplementary Figure S4), and indicated that expression trends identified in the present RNA-seq experiment were reproducible across inoculation experiments.

**FIGURE 8:**
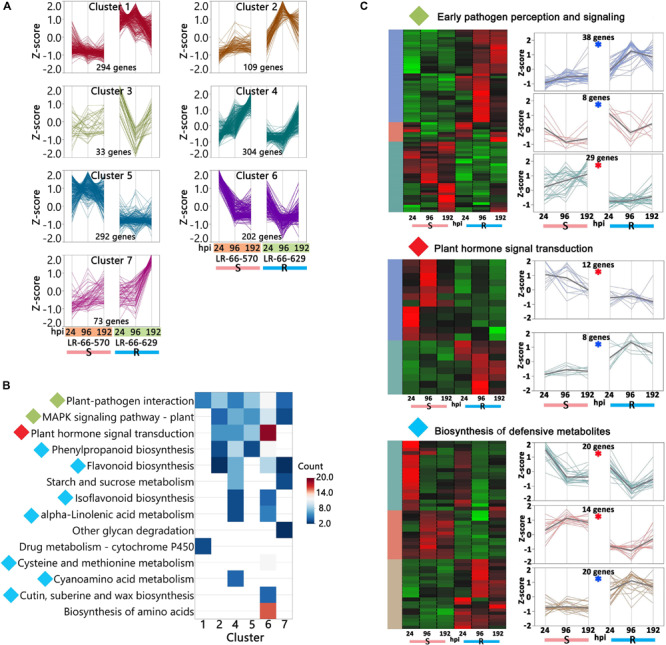
Analysis of differentially expressed genes (DEGs) between the susceptible *Lens ervoides* RIL LR-66-570 and the resistant RIL LR-66-629 after inoculation with *Ascochyta lentis*. **(A)** Hierarchical clustering (Ward’s method) analysis revealing seven clusters containing 1,307 DEGs. **(B)** Significant KEGG terms (adjusted *P* < 0.05) enriched for six clusters. No significant KEGG term was retrieved for cluster 3. KEGG terms with diamonds to the left of name were recognized as possible pathways that influenced *A. lentis* defense, where green indicated “Early pathogen perception and signaling,” red “Plant hormone signal transduction” and cyan “Biosynthesis of defensive metabolites.” **(C)** Identification of putative DEGs that may confer *A. lentis* susceptibility or resistance. Hierarchically clustering (Ward’s method) was performed for those genes involved in “Early pathogen perception and signaling,” “Plant hormone signal transduction” and “Biosynthesis of defensive metabolites.” Clusters with blue asterisk consisted of genes that were upregulated in the resistant RIL LR-66-629, those with red asterisk were upregulated in the susceptible RIL LR-66-570.

We then used hierarchical clustering and the KEGG enrichment methods to analyze these 1,307 DEGs (Supplementary Table S6). DEGs could be differentiated into seven expression clusters and were significantly (adjusted *P*-value < 0.05) enriched in 14 pathways (Figures 8A,B). Among them, eight pathways were commonly enriched in multiple clusters, including “Plant-pathogen interaction,” “MAPK signaling pathway – plant,” “Plant hormone signal transduction,” “Phenylpropanoid biosynthesis,” “Starch and sucrose metabolism,” “Isoflavonoid biosynthesis,” “alpha-Linolenic acid metabolism,” and “Flavonoid biosynthesis.” These results indicated that the genes within these pathways displayed a variety of expression patterns and play complex roles in plant disease responses. The other six pathways, “Other glycan degradation,” “Drug metabolism,” “Cysteine and methionine metabolism,” “Cyanoamino acid metabolism,” “Cutin, suberine and wax biosynthesis,” and “Biosynthesis of amino acids,” were only enriched in one cluster and seemed to respond to *A. lentis* infection in a simpler manner. Based on current knowledge, the nine pathways “Plant-pathogen interaction,” “MAPK signaling pathway-plant,” “Plant hormone signal transduction,” “Phenylpropanoid biosynthesis,” “Flavonoid biosynthesis,” “Isoflavonoid biosynthesis,” “Cyanamino acid metabolism,” “Cutin, suberine and wax biosynthesis,” and “alpha-Linolenic acid metabolism” were most likely involved in plant defense responses (Meng and Zhang, 2013; Piasecka et al., 2015). After plants sense the invading pathogens, pathogen reception and mitogen-activated protein kinase (MAPK) cascades are the earliest perception and signaling events that trigger a chain of downstream reactions in hormone regulation and signaling, and biosynthesis of defensive metabolites (Meng and Zhang, 2013). Therefore, we grouped “Plant-pathogen interaction” and “MAPK signaling pathway-plant” into an “Early pathogen perception and signaling” category, kept “Plant hormone signal transduction” as an independent category, and merged “Phenylpropanoid biosynthesis,” “Flavonoid biosynthesis,” “Isoflavonoid biosynthesis,” “alpha-Linolenic acid metabolism,” “Cysteine and methionine metabolism,” “Cyanamino acid metabolism,” and “Cutin, suberine and wax biosynthesis” into a “Biosynthesis of defensive metabolites” category. The DEGs that were either upregulated in LR-66-570 or LR-66-629 in those categories were then selected and subjected to hierarchically clustering analysis (Figure 8C). According to their expression patterns, it was hypothesized that 74 genes (indicated by a blue asterisk in Figure 8C) that were highly expressed in the resistant RIL LR-66-629 were involved in *A. lentis* resistance, whereas another 75 genes (indicated by a red asterisk in Figure 8C) which displayed a reverse trend, were assumed to be associate with *A. lentis* susceptibility.

**FIGURE 9:**
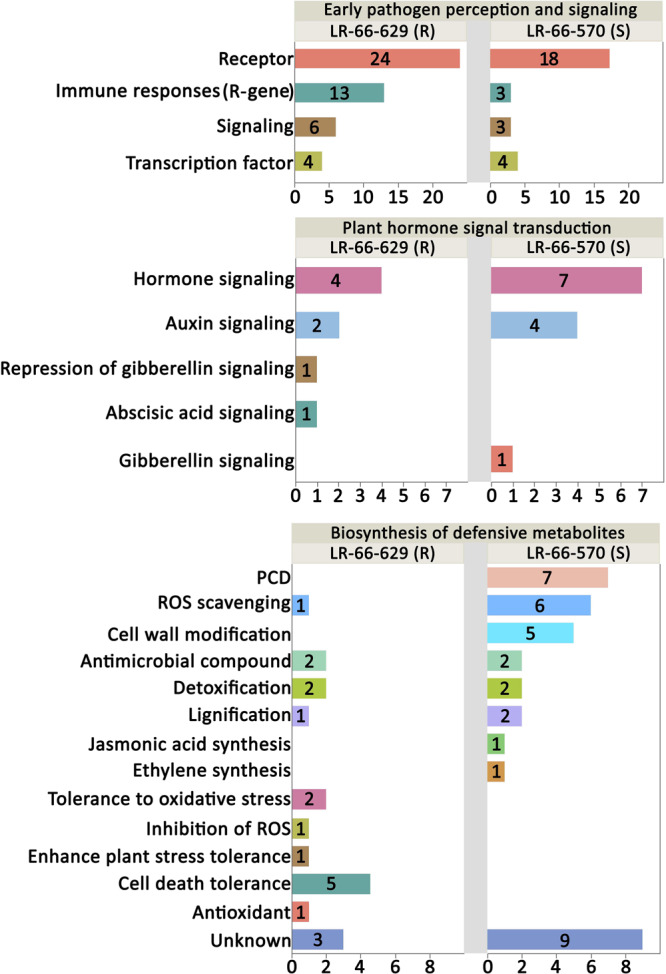
Functional analysis of putative resistance and susceptibility genes in *Lens ervoides* against *Ascochyta lentis*. Putative resistance and susceptibility genes were searched against the published literatures to understand their functions in plant disease defense system. The vertical axis indicates the functional category and the horizontal axis represents the number of genes per category.

Based on published data, 70 of the 74 genes upregulating in the resistant LR-66-629 and 66 of the 75 genes upregulating in the susceptible LR-66-570 have a documented function in plant defense responses (Figure 9 and Supplementary Table S6). In the “Plant pathogen interaction and signaling” category, there were more genes upregulating for immune responses (R-genes) and signaling in LR-66-629 than LR-66-570, suggesting that there were more genes in LR-66-629 actively interacting with, or responding to *A. lentis* during the infection and pathogen perception process. In the “Plant hormone signal and transduction” category, results showed that LR-66-629 repressed gibberellic acid (GA) signaling, while LR-66-570 promoted GA signaling, indicating that GA signaling may affect *A. lentis* resistance. When considering genes in the “Biosynthesis of secondary metabolites” category, HR appeared to be promoted in the susceptible LR-66-570 through elevated PCD, ROS scavenging and cell wall modification. In contrast, HR seemed suppressed in LR-66-629 via antioxidant synthesis, ROS inhibition, and oxidative stress and cell death tolerances.

## Discussion

The severity of symptoms caused by *A. lentis* on lentil depends on how soon the pathogen germinates, penetrates, and extends into the host tissue, and how extensively it can invade host cells. From a histopathological point of view, *A. lentis* exhibited slower germination on, and reduced penetration and colonization into the resistant LR-66-629 than into the susceptible LR-66-570. These observations were supported by qPCR estimation of fungal biomass, revealing higher fungal biomass in LR-66-570, particularly after 96 hpi when fungal biomass significantly increased along with the aggravated leaf necrosis. A reduction in conidial germination, germ tube elongation, appressorium formation, colonization and lesion size were also observed in other resistant lentil or pea accessions which were infected by *A. lentis* and *Peyronellea pinodes* (formerly *Didymella pinodes*, anamorph *A. pinodes*), respectively, (Carrillo et al., 2013, 2014; Dadu et al., 2018). These histopathological studies agreed that an initial delay in colonization followed by a subsequent restriction of *in planta* fungal growth and host necrosis are resistant mechanisms against ascochyta blight. In addition, plant cell viability tests indicated that the transition between intercellular colonization (without obvious specialized structures characteristic for hemibiotrophs) and necrotrophy likely occurred at around 96 hpi in LR-66-629 and probably much earlier than that in LR-66-570. This agrees with Sari et al. (2017) who found delayed necrotrophic growth of *A. lentis* on the resistant *L. culinaris* subsp. *culinaris* cultivar CDC Robin compared to the susceptible genotypes Eston and 964a-46, though the exact timing was different. These results suggest that an early necrotrophic phase is an important determinant for aggressive growth of *A. lentis* and susceptibility of the host. In contrast, Sambasivam et al. (2017) reported that the promotion of cell death delayed the colonization of *A. lentis* in hosts, proposing induction of cell death as a defense mechanism in the resistant lentil genotype ILL7537. More intriguingly, Carrillo et al. (2013, 2014) observed an association between aggravated epidermal cell death and reduced leaf lesions in the resistant pea accession after challenge by *P. pinodes.* These could be indicative for the presence of complex resistance mechanisms among pulse crop species.

Analysis of host and pathogen sequencing data by PCA allowed us to capture simultaneous changes in the host and pathogen transcriptomes. For the hosts, results showed that the challenge by *A. lentis* induced dramatic transcriptome reprogramming in both RILs. This is a commonly observed phenomenon in diseased plant tissues as pathogen attack activates the plant immune response that involves the reprogramming of a broad range of pathways and a large number of genes (Xin et al., 2012; Jain et al., 2016; Nobori et al., 2018). Transcriptional behaviors of the two RILs varied between early (24 hpi) and late infection (96 and 192 hpi) stages, manifesting a clear temporal dynamic. For the pathogen, diverging *A. lentis* transcriptional behaviors between 96 and 192 hpi were clearly observed in LR-66-629 but not in LR-66-570, confirming the facts that pathogen transcriptomes were shaped by the immune systems of their hosts. Supported by histopathological and fungal biomass data, we hypothesize that the distinct transcriptional profile of *A. lentis* in LR-66-629 at 96 hpi represents the transition phase switching from symptomless colonization (incubation period) to necrotrophy. It is highly likely that this delayed switch to necrotrophy of *A. lentis* in LR-66-629 was due to the disease resistance mechanisms in this host.

In the transcriptome of *A. lentis*, 36 secretory DEGs were identified for further analyses to gain more insight into how *A. lentis* invaded the two RILs. Considering that a large proportion of secretory genes were annotated to encode cell wall degrading enzymes (CWDEs), pathogenicity of *A. lentis* appears to largely depend on its ability to overcome plant cell wall barriers. As expected, the majority of cell wall degrading genes were expressed by the pathogen at a higher level in the susceptible LR-66-570 than in LR-66-629 at 96 or 192 hpi, which probably contributed to the aggravated plant cell damages caused by *A. lentis* in LR-66-570 after 96 hpi. The upregulation of these CWDEs can serve as indicators of the necrotrophic phase of *A. lentis*, similar to the massive activation of CWDEs characteristic for the necrotrophic phase of *Colletotrichum gloeosporioides* (Alkan et al., 2015). Meanwhile, we also identified four putative effector genes, three of which were annotated as CWDEs, indicating that those CWDEs were involved in the activation of plant immune responses. However, how those effectors interacted with the host immune systems cannot be answered in this study. Further study of these *A. lentis* effectors at the biological level is warranted to elucidate their roles in plant-pathogen interaction.

Analyzing a consensus co-expression network between RILs, we were able to show that the majority of genes in the two RILs co-expressed in a similar manner during the infection process, regardless of the level of resistance. Some of the genes employed similarly, in both RILs were involved in disease-defense related pathways, such as “Plant-pathogen interaction,” “MAPK signaling pathway – plant,” and “Flavonoid biosynthesis.” These genes may be involved in PTI, which is a non-specific defense mechanism that commonly occurs in all plant species after challenge by a pathogen (Lee et al., 2017). It was also observed that both RILs upregulated genes in “mRNA surveillance pathways,” “RNA degradation,” and “Ubiquitin mediated proteolysis” pathways after challenge with *A. lentis*, which suggests an elevated mRNA and protein decay in infected leaves. It has been extensively reported that the promotion of those pathways is closely associated with the aggregation of aberrant mRNA and misfolded proteins during stress conditions (He and Kermode, 2010; Coll et al., 2011; Kelley and Estelle, 2012; Mittler, 2017). These results indicate that *A. lentis* infection disrupts the normal transcription and translation in infected cells in both RILs. In addition, we also observed that genes in the two DNA synthesis pathways “DNA replication” and “Pyrimidine metabolism,” and the DNA-repair pathway “Nucleotide excision repair” were downregulated after pathogen invasion. Reports of pathogen-induced host DNA damage were recently published on *Phytophthora infestans-*infected potatoes (Song and Bent, 2014), *Pseudomonas syringae*-infected tomatoes (Song and Bent, 2014), and *Fusarium solani*-infected peas (Hadwiger and Tanaka, 2017). This accumulating evidence for a pathogen-induced damage of DNA may indicate that *A. lentis* is weakening the host’s defense responses by damaging DNA, disrupting DNA synthesis and suppressing the host’s DNA-repair system.

Network comparison on the basis of gene connectivity led to the interesting finding of variation in co-expression relationships between the susceptible RIL LR-66-570 and the resistant RIL LR-66-629. The differences in gene co-expression involved in “lipid localization and sulfur process, and cellular responses to nutrients and stimuluses” suggest that the two RILs possess different regulatory mechanisms for those biological processes. During the *A. lentis* infection process, the downregulation of lipid localization and sulfur process concurred with the upregulation of cellular responses to nutrients and extracellular stimuli. These results agreed with previous observations that plants could sacrifice certain metabolic pathways to compensate for the energy and nutrients consumed to support plant defense responses (Bilgin et al., 2010; Rojas et al., 2014). Accumulating evidence suggests that, both, lipids and sulfur are important plant regulators, with the former primarily functioning in modulating cell walls, signal transduction, protein trafficking, and hormone synthesis, and the latter responding to the biosynthesis of sulfur-containing amino acids such as methionine, cysteine and homocysteine, which are essential building blocks for a series of cofactors, transmembrane receptors and secondary metabolites (Shah, 2005; Alvarez et al., 2012; Saint-Macary et al., 2015). It was shown that the trade-off between those biological processes during pathogen attack could result in the alterations of cell wall composition, hormone signaling, ROS or SA levels which were important determinants in plant defense responses (Gao et al., 2017). In addition, a previous proteomic study showed that superior ascochyta blight resistance of a pea cultivar could be due to the higher expression of proteins involved in energetic and amino acid metabolism (Castillejo et al., 2010). These results seem to propose that the better allocation of energy and other biological functions contributed to enhanced *A. lentil* resistance.

At the single-gene level, DGE analysis revealed varying transcriptional responses between RILs. When plants are infected by pathogens, the perception and early signaling is mediated by transmembrane protein kinases such as leucine-rich repeat receptor-like kinases (LRR-RKs), cysteine-rich receptor-like kinases (CRKs) and MAP kinase kinase kinase (MAP3K). The early perception of *A. lentis* may be a resistance mechanism in lentil, as was proposed by Khorramdelazad et al. (2019) based on higher expression levels of LRR-RKs that triggered the earlier recognition of *A. lentis* in the resistant *L. culinaris* accession. However, this conclusion cannot be drawn from the current study because both, LR-66-629 and LR-66-570, have their own sets of upregulated LRR-RKs. A more plausible explanation can be found in the most recent studies where LRR-RK homologs were found to work interactively as a perception network, and variations in networks could result in different phenotypes (Huang et al., 2018; Smakowska-Luzan et al., 2018). Since different receptor homologs that responded to *A. lentis* infection were observed in the two RILs, we propose that LR-66-629 and LR-66-570 likely vary in their perception networks which may influence their response time to *A. lentis*. To date, detailed studies of perception networks with respect to pathogen stimuli have been lacking, but it will be of high interest to elucidate this machinery in future research. The genes encoding nucleotide binding site-leucine-rich repeat (NBS-LRR), which are referred to as resistance genes (R-genes), are considered the main contributors in elicitor reception, signal transduction and amplification (Bonardi et al., 2011). Studies have shown that R-genes can enhance resistance against necrotrophs by inducing an appropriate level of ROS that promotes the production of antimicrobial compounds (Quan et al., 2008; Zhu et al., 2017). So far, a variety of R-genes governing resistances against ascochyta blight have been reported in a *L. culinaris* accession and several chickpea accessions (Coram and Pang, 2006; Khorramdelazad et al., 2019; Zhou et al., 2019). As the number of upregulated R-genes in LR-66-629 was higher than that in LR-66-570, we hypothesize that some of these R-genes upregulated in the former may contribute to its enhanced *A. lentis* resistance. However, for other R-gene homologs upregulated in LR-66-570, it is still unclear whether they contributed to resistance or susceptibility toward *A. lentis*, as R-gene and HR-induced host susceptibility have been found in multiple plant-necrotroph systems (El Oirdi and Bouarab, 2007; Lorang et al., 2007; Hammond-Kosack and Rudd, 2008). Regarding hormone regulation, previous results showed that gibberellin signaling was negatively associated with necrotrophic pathogen resistance (Mengiste, 2012), which agrees with the present observation of a GA signaling repressor (Lc08554: GRAS family transcription factor) upregulated in the resistant RIL LR-66-629 and a GA signaling activator (Lc29377: Xyloglucan Endotransglucosylase) upregulated in the susceptible RIL LR-66-570. Available data from other studies have well documented that defense metabolites are significantly involved in disease defense responses by modulating the phytoalexin and hormone synthesis, signaling, ROS, and PCD (Pusztahelyi et al., 2015). In this study, *A. lentis* infection induced upregulation of antimicrobial and detoxification genes in LR-66-629 and LR-66-570, and both upregulated the same number of genes involved in these processes. Although different genes or homologs were upregulated in the two RILs, we were unable to determine if enhanced *A. lentis* resistance in LR-66-629 was affected by antimicrobial and detoxification activities as it was impossible to identify whether and which of those genes or homologs contributed more to enhanced resistance. Previous research identified a variety of genes regulating defense metabolites that were found to confer enhanced resistance against ascochyta blight in several pea and chickpea accessions (Coram and Pang, 2006; Fondevilla et al., 2014; Leo et al., 2016; Castillejo et al., 2020). In the current study, we found that the most diverging defense strategies between RILs centered primarily around ROS, cell wall modification and PCD which were mostly promoted in LR-66-570, but attenuated in LR-66-629. The promotion of ROS and PCD was also reported as susceptible factors in of the interaction of *L. ervoides* and *Stemphylium botryosum*, another necrotrophic pathogen of lentil (Cao et al., 2019). These results indicated the anti-HR machinery is not only essential for *L. ervoides* resistance against *A. lentis*, but may be also required for resistance against other necrotrophic pathogens. With respect to cell wall modifications, we found that this was only promoted in LR-66-570, and therefore consider this to be associated with susceptibility rather than resistance, as was reported for ascochyta blight resistance in several pea accessions (Fondevilla et al., 2014; Castillejo et al., 2020). These conflicting results reflect the high complexity of cell wall-related resistance as cell wall remodeling may interfere the spread of pathogen or can be taken advantage of by the pathogen to facilitate their entry into tissue (Bellincampi et al., 2014).

## Materials and Methods

### Plant Materials

Highly resistant RIL LR-66-629 (2.5% ascochyta blight severity; Bhadauria et al., 2017) and moderately susceptible RIL LR-66-570 (41% ascochyta blight severity; Bhadauria et al., 2017) were transgressive segregants selected from an F_9_ recombinant inbred population derived from a cross between the *L. ervoides* accessions L01-827A and IG 72815. A previous study showed that L01-827A and IG 72815 were both partially resistant to *A. lentis* and were therefore regarded as important sources for *A. lentis* resistance genes (Bhadauria et al., 2017). Scarified seeds of LR-66-629 and LR-66-570 were sowed in 10 cm square pots with nine seeds per pot. All plants were maintained in a growth chamber with a constant temperature of 21°C and a photoperiod of 16 h. When plants were 24 days old, they were thinned to four vigorous seedlings per pot for subsequent inoculation experiments.

### Inoculation Procedure and Experimental Design

The inoculum was prepared from a monoconidial culture of *A. lentis* isolate AL-61 which is an aggressive isolate collected from Landis, Saskatchewan, Canada. For spore revitalization, the cryopreserved *A. lentis* spores were cultured on 50% oatmeal agar plates [30 g oatmeal (Quick Oats, Quaker Oats Co., Chicago, IL, United States), 8.8 g agar (Difco, BD^®^, Sparks Glencoe, MD, United States), 1000 mL sterile H_2_O] at room temperature for 7 days. Conidia were dislodged with sterile distilled water and the spore suspension was adjusted to 5 × 10^5^ conidia mL^–1^. For each pot, the prepared spore suspension was sprayed onto plants until runoff, which was equivalent to an approximate application rate of 2.5 mL plant^–1^. The same amount of water was used for mock inoculation of control plants. Four plants within a pot were pooled to generate one biological replicate. The experimental design followed a completely randomized design with three replicates. After inoculation, plants were incubated in a misting tent at 100% humidity for 48 h to ensure spore germination and infection. Plants were removed from the misting tent 48 h post inoculation (hpi), and enclosed in translucent plastic bags to maintain the appropriate levels of humidity. Inoculated leaves were collected at 0, 6, 12, 24, 48, 72, 96, 144, 196, 240, 288, and 336 hpi and immediately frozen in liquid nitrogen for future use.

### Histopathological Observation

Six leaves per biological replicate for each of the two RILs were collected at 6, 12, 24, 48, 72, 96, 144, 192, and 240 hpi and fixed immediately in 5 ml tubes filled with CMAA fixative solution (60% methanol, 30% chloroform, and 10% acetic acid) at room temperature for at least 24 h until the leaflets were cleared. After fixation treatment, all leaflets were stored in 95% ethanol at room temperature. Prior to use, these leaflets were rehydrated through decreasing ethanol concentrations of 70% (1 h), 50% (1.5 h), and 30% (1.5 h). Leaflets were stained overnight using 0.05% trypan blue. Stained leaflets were mounted on microscope slides in a droplet of 50% glycerol and slides were examined under bright field of a Zeiss Axioskop 40 microscope (Carl Zeiss, Göttingen, Germany). Photos were taken using a Pixelink A686C camera (Pixelink, Rochester, NY, United States) and Zeiss AxioVision software (Carl Zeiss). To quantitatively assess development of *A. lentis* during the incubation process, the numbers of germinated conidia per 50 conidia, appressoria formed per 25 germinated conidia, and pycnidia were recorded for each leaflet. To assess leaf necrosis, the percentage area of dead tissue per leaflet was visually estimated. Mean separations of fungal growth parameters between RILs were performed using Student’s *t*-test at the significant level of *P* < 0.05. Plant cell viability tests for two RILs were performed as proposed by Sari et al. (2017) with some modifications (W.G.H.M. Hendriks and Y. Wei, Deptarment of Biology, University of Saskatchewan, personal communication) using Neutral Red and the adaxial epidermal cells were then assessed with a Zeiss Axioskop 40 microscope.

### Determination of Relative Fungal Growth Using qPCR

*Lens ervoides* elongation factor (LcEF1-α) and the *A. lentis* β-tubulin (TL-1) were amplified from DNA extracted from the two RILs infected with *A. lentis* using gene-specific primers (Supplementary Table S1). DNA was extracted with the SDS method (Goldenberger et al., 1995), quantified with a spectrophotometer (NanoDrop^TM^ 8000, Thermo Scientific, Waltham, United States) and adjusted to a concentration of 25 ng μl^–1^. Each qPCR reaction contained 2 μl DNA template, 5 μl SYBR^®^ Green (Thermo Scientific), 0.2 μl of each 10 μM forward and reverse primers, and 2.6 μl distilled water. The qPCR amplifications were performed in a QuantStudio^TM^ 3 System (Applied Biosystems Inc., Foster City, CA, United States) using a fast-run program with default settings. The relative fungal biomass was calculated as the ratio of gDNA amplified by the TL-1 primer over that amplified by the LcEF1-α primer using the formula *gDNA*_*F*_ = 2^−(*CT*_*TL*−1_−*CT*_*LC*_*EF*_1−α_)^, according to the criteria used by Horevaj et al. (2011).

### RNA Extraction, Quality Control, Sequencing and Raw Data Processing

Based on the results from fungal biomass quantification through qPCR and histopathological observations, mock- and *A. lentis*-inoculated plant samples of LR-66-570 and LR-66-629 collected at 24, 96, and 192 hpi were selected for further analyses, using three biological replicates each for a total of 36 samples. Total RNA was isolated from the frozen leaves using the RNeasy Plant Mini Kit (Qiagen Company, Hilden, Germany). RNA quality and concentration were pre-determined on an Agilent 2100 Bioanalyzer (Agilent Technologies, Santa Clara, CA, United States) before processing for library construction using TruSeq Stranded Total RNA (Illumina, Inc., San Diego, CA, United States). The 36 libraries were pooled into two lanes to generate clusters using the TruSeq PE Cluster Kit V4-cBot-HS (Illumina) and then fed into a HiSeq 2500 system using TruSeq SBS KIT-HS V4 (Illumina) for 125 bp pair-ended sequencing at Canada’s Michael Smith Genome Sciences Center (BCGSC, Vancouver, BC, Canada). The returned raw reads were filtered with Trimmomatic (version 0.36) (Bolger et al., 2014) to remove adaptors and low quality reads specifying the following parameters: TruSeq3-PE-2.fa:2:30:10, leading:3, trailing:3, slidingwindow:4:15 and minlen:36, resulting in 15 to 27 million reads per sample.

### Separation of Plant- and Pathogen-Specific Reads

Reads that passed quality control were then mapped against the *L. culinaris* reference genome V1.2 (Bett et al., 2016) using STAR (version 2.6.1a, default settings) (Dobin et al., 2013). To enrich reads belonging to *A. lentis*, those reads that could not be mapped to the *L. culinaris* genome were identified and processed. First, unmapped reads from mock-inoculated plants at all time points were pooled together and *de novo* assembled (Trinity version 2.84, default settings) (Grabherr et al., 2011) into a so-called “leftover transcriptome.” Then, the unmapped reads from *A*. *lentis*-inoculated samples were aligned to this “leftover transcriptome.” The resulting unaligned reads were assumed to be from *A*. *lentis*. Those unaligned reads were again filtered with the help of the *L. culinaris* genome and “leftover transcriptome” to remove any possible matches using Fastq_screen (version 0.11.4) (Wingett and Andrew, 2018). Only unmatched reads were considered as *A. lentis* reads for subsequent *de novo* transcriptome assembly.

### *De novo* Assembly, Assessment, and Annotation of *A. lentis* Transcriptome

The enriched pathogen reads from three time points were pooled together and *de novo* assembled into an *A. lentis* transcriptome using the Trinity software (default settings). The software BUSCO (version 3.0.2) was used to assess the completeness of the transcriptome (Simão et al., 2015). The general statistics of assembly were obtained by using “TrinityStats.pl” script affiliated with Trinity.

After clearing of transcriptomic redundancies with the software CD-hit (version 4.81) (Fu et al., 2012), long ORFs (>100 amino acids) were extracted from the pathogen transcriptome with TransDecoder (version 5.5.0, default settings) (Haas et al., 2013). Possible homologs of these ORFs were identified with BlastP (e-value = 1e-5) using the fungi RefSeq database in Diamond (version 0.9.8) (Buchflink et al., 2015) and hmmscan against Pfam database in HMMER (version 3.1b2) (Wheeler and Eddy, 2013). Homologs with known functions were retained even if their coding sequence (CDS) were not significantly predicted by TransDecoder in the subsequent step.

For the *A. lentis* secretome predictions, predicted proteins were submitted to SignalP (version 3.0) (Bendtsen et al., 2004) and TargetP (version 1.1, location = “Secreted”) (Emanuelsson et al., 2000) for signal peptide motifs and protein localization predictions, respectively. The software TMHMM (version 2.0) (Krogh et al., 2001) was then used to identify the possible transmembrane domain(s) for each of the predicted proteins (predicted signal peptides were removed). Predicted signal proteins were scanned for the presence of the terminal motifs “KDEL” or “HDEL,” and were tagged when present, as these motifs are knowns to be ER-retention signals. Only those signal proteins without transmembrane domain or ER-retention motif were considered as *A*. *lentis* secretory proteins. Thereafter, the sequences of all predicted secretory proteins were submitted for effector prediction to EffectorP 2.0^1^. Subsequently, the *A. lentis* secretome was annotated with InterProScan software (version 5.35-74.0, default settings) (Jones et al., 2014) using a series of databases of functional domains (PANTHER, Pfam, Coils, Gene3D, SUPERFAMILY, SMART, PIRSF, and PRINTS) and BlastP (e-value = 1e-5) employing the fungi RefSeq database.

### Gene Expression Quantification, Normalization, and Visualization

Since alignments of reads were different between *L. ervoides* and *A. lentis*, different approaches were adopted to quantify their abundance. For *L. ervoides*, gene expression was quantified based on raw counts using -quantMode during the mapping process in STAR. Gene expression for *A. lentis* was assessed in the form of transcripts per kilobase million (TPM) on the “Trinity gene” level by using the “align_and_estimate_abundance.pl” script (RSEM method) provided by Trinity.

The gene expression normalization among samples in *L. ervoides* or *A. lentis* were performed using the trimmed mean of m-values (TMM) in JMP Genomics 8.0 (JMP Genomics^®^, SAS Institute). The overall gene expression variability among samples was visualized using principal component analysis (PCA) in the same software.

### Weighed Gene Co-expression Network Analysis (WGCNA) of *L. ervoides* Genes

Gene co-expression network analysis was implemented in the package WGCNA (version 1.64-1) (Langfelder and Horvath, 2007) in R version 3.5.0. Firstly, we examined the consensus gene co-expression network between the susceptible RIL LR-66-570 and the resistant RIL LR-66-629. A consensus network describes the shared gene co-expression relationships among multiple individual networks. After removing genes with low expression (TMM < 25), a total of 16,786 genes were used for constructing a consensus network and generating modules by using the blockwiseConsensusmodules function in WGCNA with the following settings: power = 9, minModuleSize = 30, deepSplit = 2, and mergeCutHeight = 0.25. To select the modules consisting of disease responsive genes, we used the number “0” to represent mock-inoculated samples and “1” for *A. lentis*-inoculated samples, and then associated those numbers with the Gene Significance value calculated for each module. The resulting significantly correlated modules with major sizes (*P* < 0.01, gene number > 1000) were processed for further analyses.

Secondly, we compared the gene co-expression relationship between RILs. To do so, we constructed a network for the resistant RIL LR-66-629 using the following parameters: TOMType = “unsigned,” power = 9, deepSplit = 2, minModuleSize = 30, and mergeCutHeight = 0.25. These network settings in LR-66-629 were then assigned to LR-66-570 for a preservation test using the default build-in function of modulePreservation. The preservation indexes of module size, Zsummary and Median rank were retrieved to assess the preservation level for each module (Langfelder et al., 2011). Selection of the hub genes and network visualization were performed with VisANT (version 5.51) (Hu et al., 2008). The genes of interest with known *Medicago truncatula* orthologs were enriched in the databases of Kyto Encyclopedia of Genes and Genomes (KEGG) or Gene Ontology (GO) using the R package clusterProfiler (Yu et al., 2012).

### Differential Gene Expression Analysis of *L. ervoides* Genes

Prior to comparing gene expression between RILs, we first performed pair-wise comparisons between mock- and *A. lentis*-inoculated samples in each RIL at 24, 96 and 192 hpi to identify those genes that significantly responded to *A. lentis* infection. The statistical analysis of mean separations was performed with DESeq2 (Anders and Huber, 2010) using the thresholds of (1) false discovery rate (FDR) < 0.05 and (2) gene expression fold change > 2 to declare the disease responsive genes. These disease responsive genes were then used for detection of differential expressed genes (DEGs) between LR-66-570 and LR-66-629. The same settings used in the previous step were applied here to declare significant DEGs. The method of hierarchical clustering (Ward’s method) was used to separate DEGs into varying expression patterns in JMP Genomics 8.0 (JMP Genomics^®^, SAS Institute). Genes projected to the corresponding *Medicago truncatula* orthologs were mapped to KEGG database through the R package clusterProfiler (Yu et al., 2012) to recognize their roles in biological pathways.

### Differential Gene Expression Analysis of *A. lentis* Genes

For *A. lentis*, TMM-normalized TPMs were submitted to JMP Genomics 8.0 for pair-wise comparisons of all pathogen samples. The thresholds of FDR < 0.05 and fold change > 2 were used to declare DEGs. The expression patterns of DEGs were visualized via a hierarchical clustering analysis and a heatmap generated by JMP Genomics 8.0.

### Quantitative PCR Validation

To verify the repeatability of the RNA-seq experiment, we performed an independent inoculation experiment following the same experimental conditions and design, and a total of 20 genes of interest and one reference gene belonging to *L. ervoides* were chosen for qPCR amplification. The functional annotation and primer sequences for those genes are shown in Supplementary Table S1. Each qPCR reaction contained 2 μl DNA template, 5 μl SYBR Green, 0.2 μl of each 10 μM forward and reverse primers, and 2.6 μl distilled water. qPCR was performed in a QuantStudio^TM^ 3 System (Applied Biosystems Inc.) using a fast-run program with default settings. The relative expression of each gene was calculated using a formula of 2^−(*CT*_*gene of interest*_−*CT*_*reference*_)^ (Livak and Schmittgen, 2001).

## Data Availability Statement

The datasets generated for this study can be found at NCBI BioProject PRJNA632712.

## Author Contributions

ZC inoculated the plants, collected the samples, conducted the biomass determination, analyzed the RNA-seq data, and drafted the manuscript. KK performed the histopathological assessment of disease progression on plants. LL assisted in all of these experiments. SB conceived and supervised the project and revised the manuscript. All authors approved the final submission.

## Conflict of Interest

The authors declare that the research was conducted in the absence of any commercial or financial relationships that could be construed as a potential conflict of interest.

## Footnotes

1 http://effectorp.csiro.au/

## Supplementary Material

FIGURE S1 Summary of read mapping of 36 libraries on the *Lens culinaris* reference genome.

FIGURE S2 Construction of co-expression relationship between the susceptible *Lens ervoides* RIL LR-66-570 and the resistant RIL LR-66-629 after inoculation with *Ascochyta lentis*. Bars represent the scaled connectivity (0 to 1) where 1 indicates 100% correlation and 0 represents no correlation for pair of modules between RILs. The value of *D* indicates the overall scaled connectivity (0 to 1), with 1 indicating 100% correlation and 0 representing no correlation for all pairs of modules between RILs.

FIGURE S3 Hub gene recognition in Orangered3 and Lightgreen networks based on differential gene co-expression analysis between *Lens ervoides* RILs LR-66-629 (resistant) and LR-66-570 (susceptible) after inoculation with *Ascochyta lentis*. A weight-cutoff value of 0.20 was used to select 10 hub genes displaying the high intramodular connectedness in both modules. The descriptions and documented defense roles of these hub genes are shown to the right of their corresponding networks.

FIGURE S4 qPCR validation of 20 DEGs between the *Lens ervoides* RIL LR-66-570 (susceptible) and LR-66-629 (resistant) after inoculation with *Ascochyta lentis*. The validated DEGs included 10 genes that were upregulated in LR-66-570 **(A)** and the other 10 genes that were upregulated in LR-66-629 **(B)** upon inoculation with *A. lentis*.

TABLE S1 Primer sequences used in RT-qPCR for fungal biomass determination and gene expression validation. **(1)** Primers used in fungal biomass determination. **(2)** Primers used for RT-qPCR validation of gene expression.

TABLE S2 *De novo* assembled amino acid sequences of *Ascochyta lentis* proteins and predicted secretome. **(1)** Amino acid sequences of 19,834 predicted *Ascochyta lentis* proteins. **(2)** Prediction information of 904 *A. lentis* secretory proteins.

TABLE S3 Expression analysis of *Ascochyta lentis* secretory genes between two *Lens ervoides* RILs. **(1)** Statistics of differential expression analysis for 36 secretory genes of *Ascochyta lentis*. **(2)** Expression table (*Z*-score) used for hierarchical clustering analysis of 36 secretory genes of *A. lentis*. **(3)** Functional classification of 36 secretory genes among clusters.

TABLE S4 Information of consensus modules generated by weighted gene co-expression analysis (WGCNA) for two *Lens ervoides* RILs. **(1)** The consensus module eigengene generated by WGCNA for two *Lens ervoides* RILs over time. **(2)** List of *L. ervoides* genes with known *Medicago truncatula* orthologs for three major consensus modules.

TABLE S5 Analysis of module preservation between two *Lens ervoides* RILs. **(1)** The module eigengene for reference network (*Lens ervoides* RIL LR-66-629) and test network (LR-66-570). **(2)** Expression table (*Z*-score) used for hierarchical clustering analysis of genes in Lightgreen and Orangered3 modules. **(3)** List of *L. ervoides* genes with known *Medicago truncatula* orthologs for Lightgreen and Orangered3 modules. **(4)** Detailed gene connection network for Lightgreen and Orangered3 modules. **(5)** Functional classification of hub genes identified in Lightgreen and Orangered3 modules.

TABLE S6 Analysis of differentially expressed genes between two *Lens ervoides* RILs. **(1)** Information of differential gene expression between *Lens ervoides* RILs LR-66-629 and LR-66-570 at 24, 96, and 192 hpi. **(2)** Expression table (Z-score) used for hierarchical clustering analysis of DEGs and information of known *Medicago truncatula* orthologs for each DEG. **(3)** Functional classification of putative genes involved in disease defense responses.
