# Supplementary figures and images for: Interactive Gene Expression Patterns of Susceptible and Resistant Lens ervoides Recombinant Inbred Lines and the Necrotroph Ascochyta lentis
Source: Front Microbiol. 2020 Jun 24;11:1259. doi: 10.3389/fmicb.2020.01259 (PMC7326948; doi:10.3389/fmicb.2020.01259)

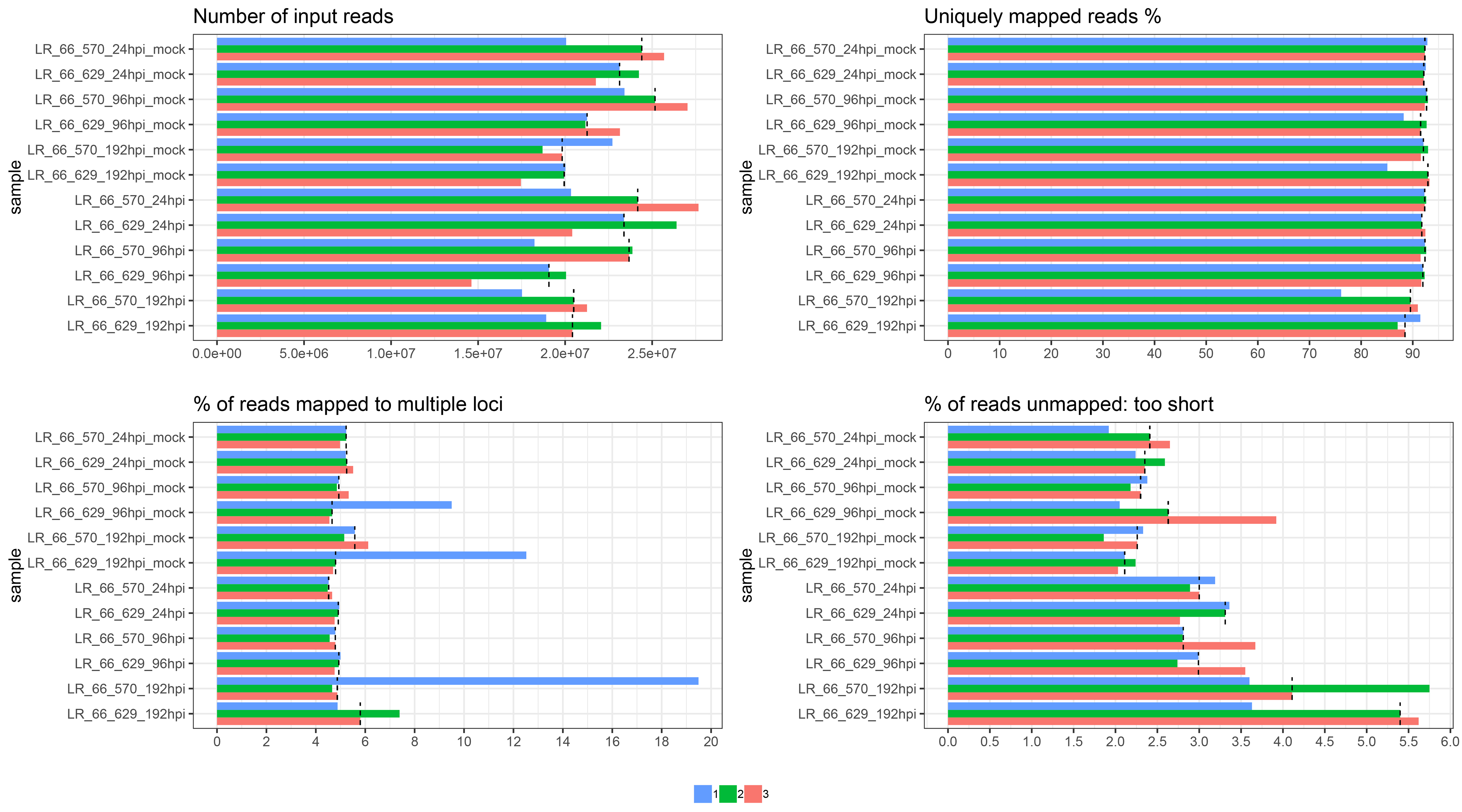

Supplement: FIGURE S1 — Summary of read mapping of 36 libraries on the Lens culinaris reference genome. [file Image_1.JPEG]

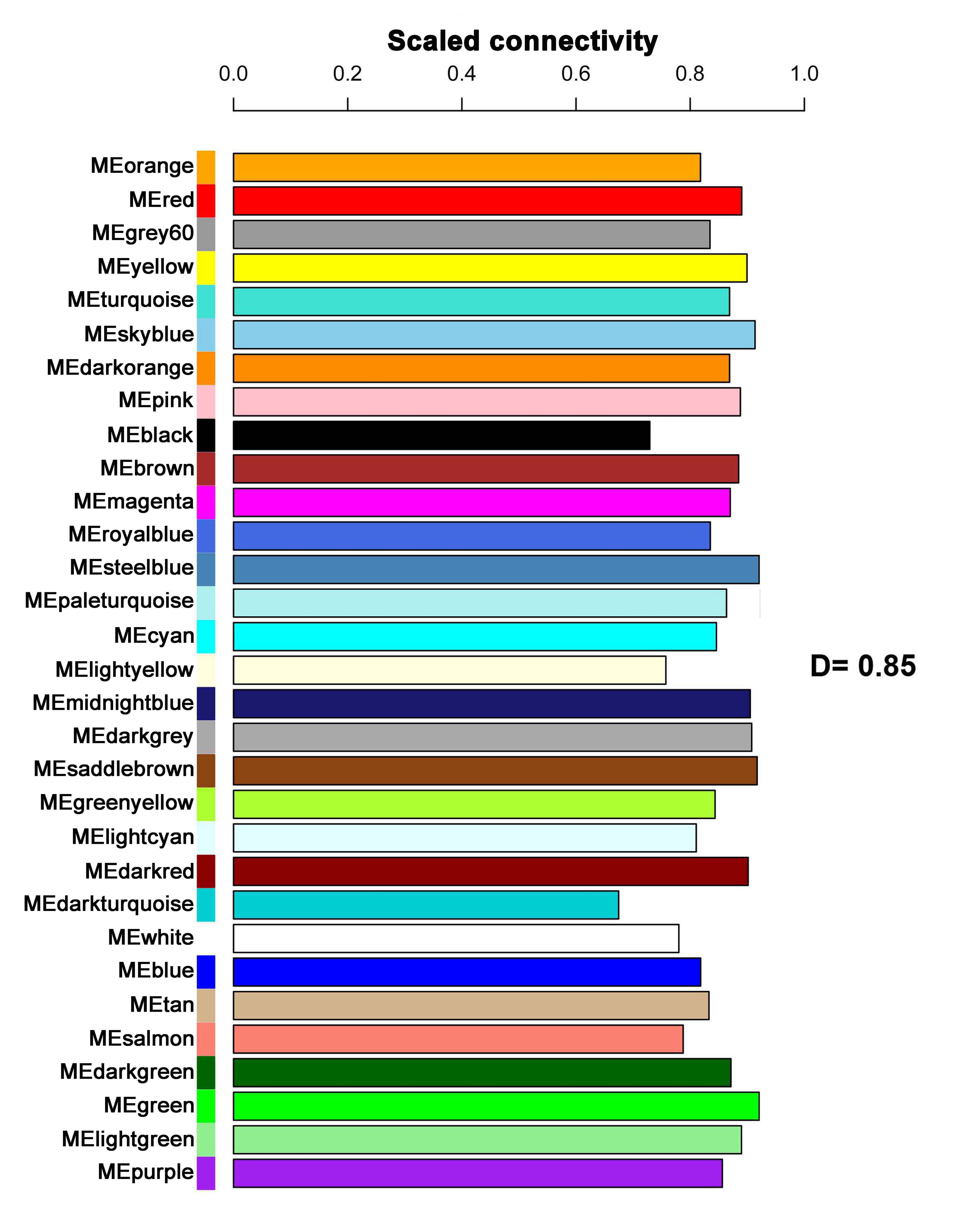

Supplement: FIGURE S2 — Construction of co-expression relationship between the susceptible Lens ervoides RIL LR-66-570 and the resistant RIL LR-66-629 after inoculation with Ascochyta lentis. Bars represent the scaled connectivity (0 to 1) where 1 indicates 100% correlation and 0 represents no correlation for pair of modules between RILs. The value of D indicates the overall scaled connectivity (0 to 1), with 1 indicating 100% correlation and 0 representing no correlation for all pairs of modules between RILs. [file Image_2.JPEG]

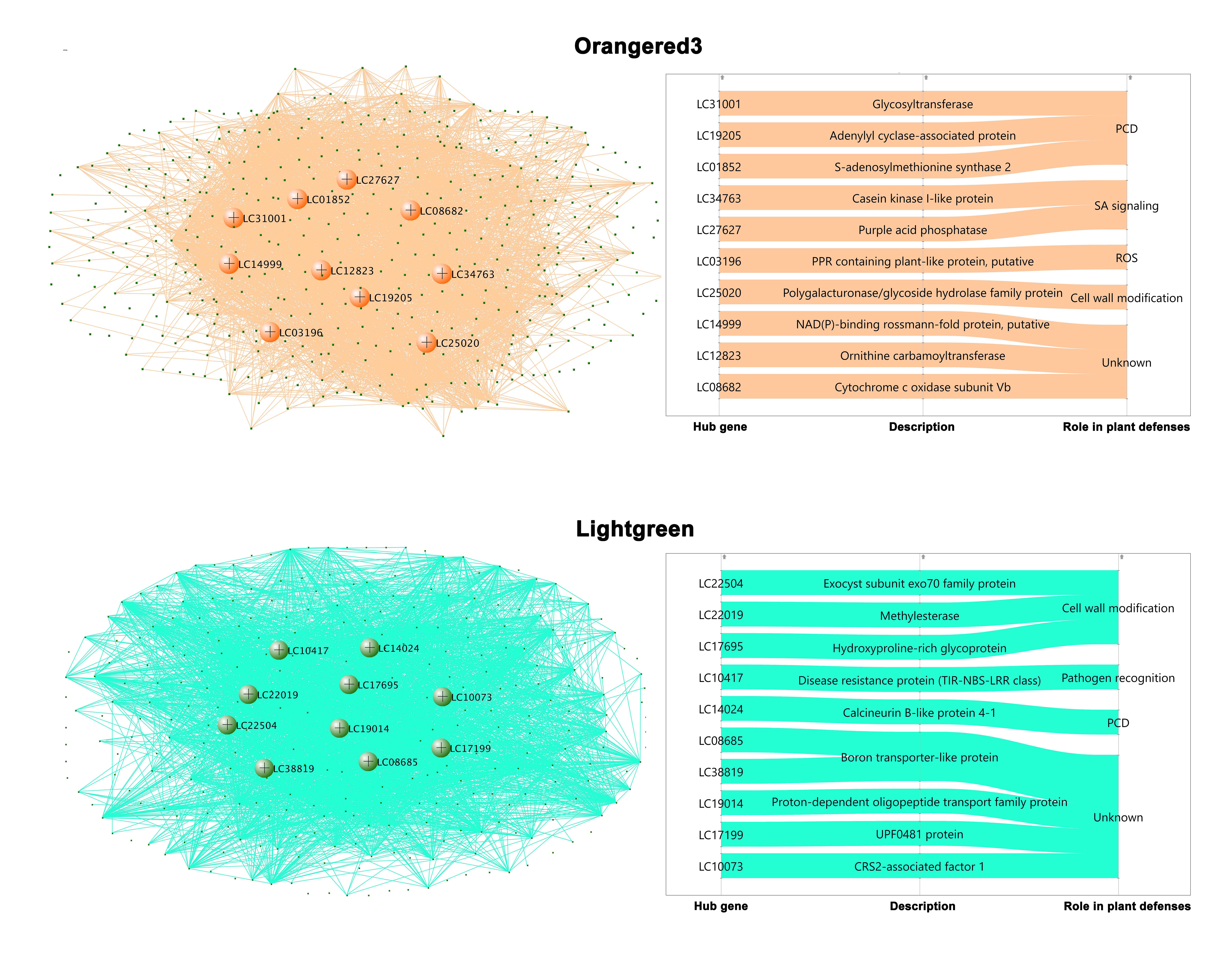

Supplement: FIGURE S3 — Hub gene recognition in Orangered3 and Lightgreen networks based on differential gene co-expression analysis between Lens ervoides RILs LR-66-629 (resistant) and LR-66-570 (susceptible) after inoculation with Ascochyta lentis. A weight-cutoff value of 0.20 was used to select 10 hub genes displaying the high intramodular connectedness in both modules. The descriptions and documented defense roles of these hub genes are shown to the right of their corresponding networks. [file Image_3.JPEG]

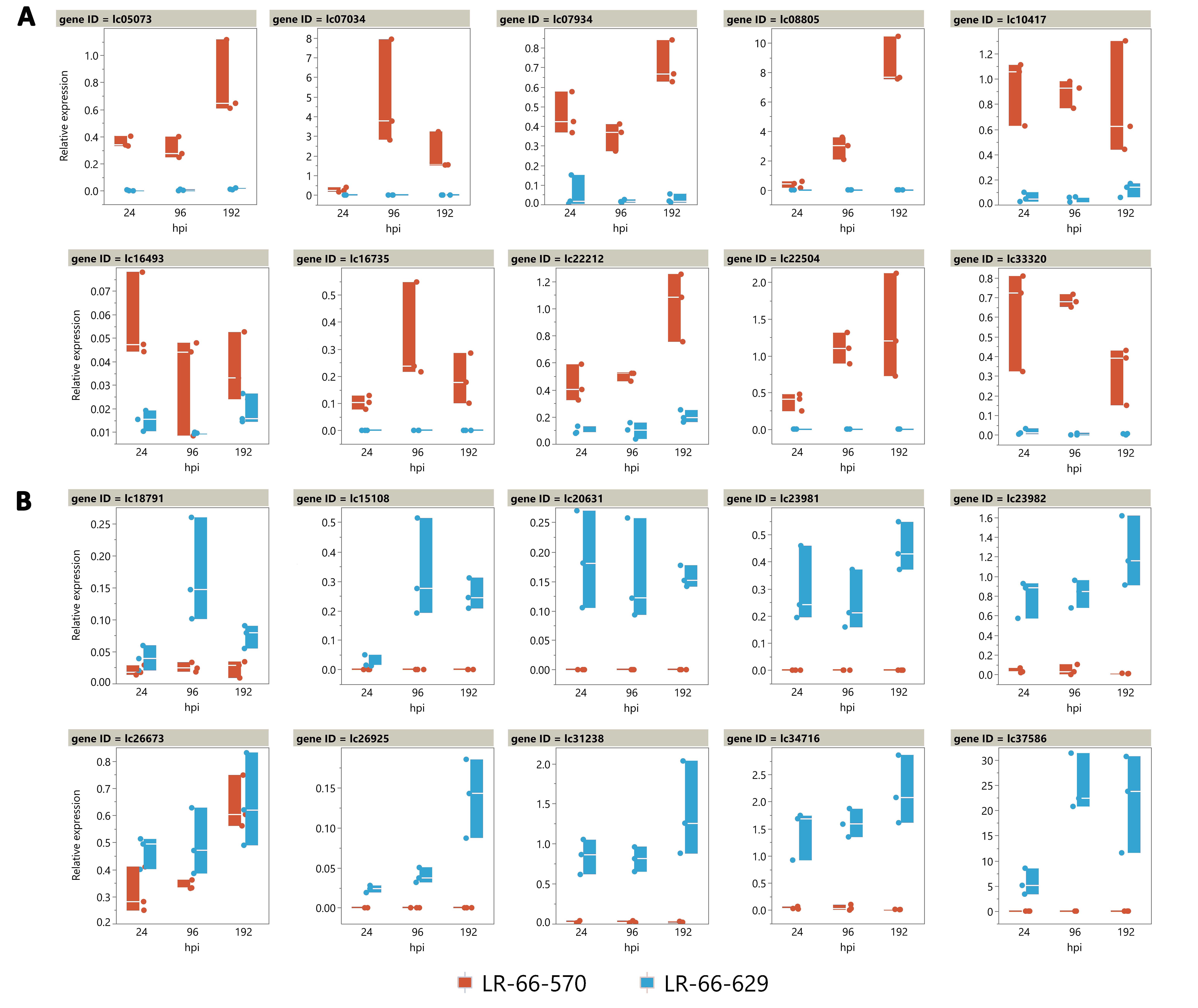

Supplement: FIGURE S4 — qPCR validation of 20 DEGs between the Lens ervoides RIL LR-66-570 (susceptible) and LR-66-629 (resistant) after inoculation with Ascochyta lentis. The validated DEGs included 10 genes that were upregulated in LR-66-570 (A) and the other 10 genes that were upregulated in LR-66-629 (B) upon inoculation with A. lentis. [file Image_4.JPEG]
